# Perspectives for New and More Efficient Multifunctional Ligands for Alzheimer′s Disease Therapy

**DOI:** 10.3390/molecules25153337

**Published:** 2020-07-23

**Authors:** Agnieszka Zagórska, Anna Jaromin

**Affiliations:** 1 Department of Medicinal Chemistry, Faculty of Pharmacy, Jagiellonian University Medical College, 30-688 Kraków, Poland; 2 Department of Lipids and Liposomes, Faculty of Biotechnology, University of Wroclaw, Wroclaw, 50-383 Wrocław, Poland; anna.jaromin@uwr.edu.pl

**Keywords:** multitarget drug discovery, MTDLs, tacrine, donepezil, AChE inhibitors, BACE-1 inhibitors, GSK-3β inhibitors

## Abstract

Despite tremendous research efforts at every level, globally, there is still a lack of effective drugs for the treatment of Alzheimer′s disease (AD). The biochemical mechanisms of this devastating neurodegenerative disease are not yet clearly understood. This review analyses the relevance of multiple ligands in drug discovery for AD as a versatile toolbox for a polypharmacological approach to AD. Herein, we highlight major targets associated with AD, ranging from acetylcholine esterase (AChE), beta-site amyloid precursor protein cleaving enzyme 1 (BACE-1), glycogen synthase kinase 3 beta (GSK-3β), *N*-methyl-d-aspartate (NMDA) receptor, monoamine oxidases (MAOs), metal ions in the brain, 5-hydroxytryptamine (5-HT) receptors, the third subtype of histamine receptor (H_3_ receptor), to phosphodiesterases (PDEs), along with a summary of their respective relationship to the disease network. In addition, a multitarget strategy for AD is presented, based on reported milestones in this area and the recent progress that has been achieved with multitargeted-directed ligands (MTDLs). Finally, the latest publications referencing the enlarged panel of new biological targets for AD related to the microglia are highlighted. However, the question of how to find meaningful combinations of targets for an MTDLs approach remains unanswered.

## 1. Introduction

In 1906, Alois Alzheimer presented his first signature case and the pathological features of the disease which, from 1910, became known as Alzheimer’s disease (AD). AD is clinically characterized by a loss of memory, the retardation of thinking and reasoning, and changes in personality and behaviours [1,2]. Nowadays, approximately 40 million people over the age of 60 suffer from AD worldwide, and the number of patients is increasing, with the perspective of cases doubling every 20 years [3]. AD is a progressive and irreversible neurological disorder occurring in the central nervous system (CNS) mainly confined within the hippocampus and the cerebral cortex, domains of the forebrain related to memory and higher cognitive functions. The histological manifestation of AD presents extracellular deposits of β-amyloid peptide (Aβ) and the intracellular formation of neurofibrillary tangles consisting of paired helical filaments of hyperphosphorylated tau protein [4,5]. AD is a complex and multifactorial disease, which means that it is influenced by a combination of multiple genes and environmental/risk factors. In the early 1990s, mutations in the genes of amyloid-beta A4 precursor protein (APP), presenilin 1 (PSEN1), and presenilin 2 (PSEN2) were determined for familial AD [6,7,8]. Presenilins are components of the γ-secretase complex which, when mutated, can affect amyloid precursor protein (APP) processing to form toxic forms of Aβ. In addition to genes, genetic risk loci for AD were determined and one of them, apolipoprotein E, type ε4 allele (APOE ε4), is associated with late-onset familial AD [9,10]. Studies on the binding of apoE (a peptide corresponding to the low-density lipoprotein receptor binding domain) to APP, showed that blocking of the interaction of apoE with *N*-terminal APP reduces Alzheimer′s-associated Aβ accumulation and tau pathologies in the brain [11]. Known risk factors include age, having a family history of AD, APOE ε4, vascular problems (heart disease, stroke, high blood pressure), diabetes, and obesity [12,13]. However, the reasons why sporadic AD occurs is still unknown. There are various descriptive hypotheses regarding the causes of sporadic AD, including the cholinergic hypothesis [13], amyloid hypothesis [14,15,16], tau propagation hypothesis [17,18], mitochondrial cascade hypothesis [19,20], calcium homeostasis hypothesis [21,22], inflammatory hypothesis [23,24,25], neurovascular hypothesis [26], metal ion hypothesis [27,28,29], and lymphatic system hypothesis [30,31]. Moreover, there are many factors that may associated with AD, such as various microbes (triggering amyloidosis), viral pathogens (*Herpesviridae* family) [32,33], decreased expression of microRNAs-107 (miRNA-107) [34,35,36,37,38] and RAS-RAF-MEK signalling pathway (atrophy of neurons) [39], regional hypometabolism [40,41], and mitochondrial dysfunction [42,43,44]. In summary, the aetiology of AD is recognized but not fully understood.

Currently, there are a total of five therapies in the clinic, and four drugs approved by the FDA for AD (Table 1), which mostly aim at restoring physiological ACh levels. The inhibition of the enzyme acetylcholinesterase (AChE), responsible for the hydrolysis of Ach, is the main biochemical mechanism of action of donepezil, rivastigmine and galantamine. In turn, memantine, through noncompetitive antagonism of the *N*-methyl-d-aspartate (NMDA) receptor, blocks current flow (especial calcium) and reduces the excitotoxic effect of glutamate [45].

Drugs only provide temporary symptomatic relief among patients with mild-to-moderate symptoms of AD, but do not provide a cure or protection. Thus, one of the largest unmet medical needs is a modifying treatment for AD. The complicated pathogenesis of AD, in association with the various descriptive hypotheses involved in the onset and development of the disease and along with the imperfect single-target drugs available, form an excellent rational basis for the implementation of multi-target strategies as part of the drug discovery pipeline against AD. The multi-target-directed ligands (MTDLs) strategy foresees the development of a single molecule able to affect several key targets/pathways, which can have a synergistic effect on the AD network, leading to superior improvement on memory and cognition. While the idea of MDTLs is simple at a glance, the rational design of a compound in which two or more pharmacophores are combined in a single molecular entity is a challenge. Herein, we highlight major targets associated with AD and collate the latest publications which have resulted in an enlarged panel of drug targets.

## 2. AD-Related Targets

**Figure 1:**
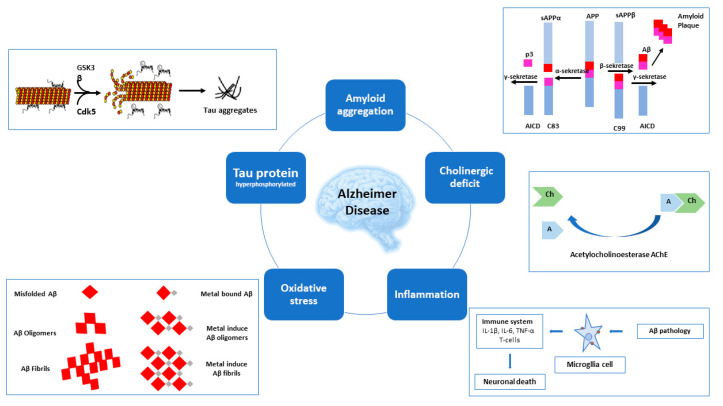
Multiple pathological pathways of AD.

Exploration of hypotheses regarding the causes of AD focus on major pathogenesis factors and pathways (Figure 1). Firstly, cholinergic deficit, which led to the discovery of the primary AD targets of acetylcholinesterase (AChE) and butyrylocholinosterase (BuChE). Next, for amyloid aggregation, where the main target is beta-secretase 1 (BACE-1), whereas for the hyperphosphorylation of tau protein, glycogen synthase kinase 3 beta (GSK-3β) and cyclin dependent kinase 5 (Cdk5) are the key targets. Increased oxidative damage and inflammation and unbalanced homeostasis of biometals in the course of AD led to the discovery of further potential targets for AD treatment.

**Figure 2:**
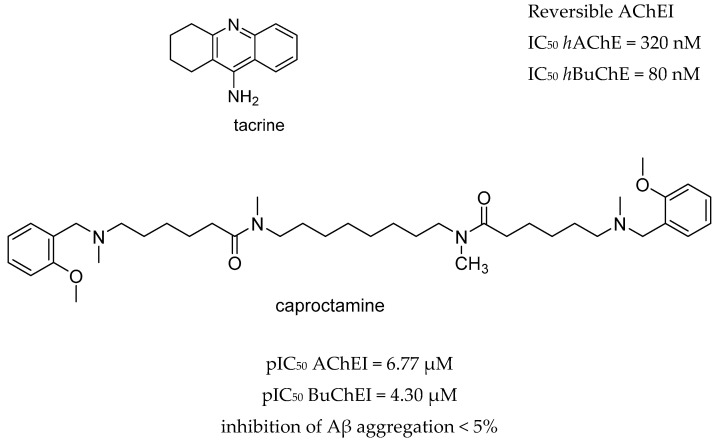
Tacrine and captopramine.

Moreover, the pathogenesis of AD involves numerous receptors, namely, (*N*-methyl-d-aspartate (NMDA), 5-hydroxytryptamine (5-HT) serotonin, the third subtype of histamine receptor (H_3_ receptor), and enzymes, namely monoaminoxidases (MAOs), and phosphodiesterases (PDEs). The first AChE inhibitor (AChEI) for AD treatment, tacrine, was approved in 1993. However it was withdrawn shortly after release due to liver toxicity [46] (Figure 2). Currently, the inhibition of AChE is a fundamental property of drugs approved by the FDA which are principally AChE inhibitors (AChEIs), such as donepezil, galantamine, and rivastigmine. Even so, tacrine and donepezil are still the targets for modifications or are used as positive controls in enzyme or pharmacological activity tests. The cholinergic hypothesis, combining ACh and AChE as a common modality is emerging as a promising approach in designing MTDLs for AD. The first clinical example of this approach was caproctamine, which was reported in 1998 as a compound with noncovalent inhibitory activity against the cholinergic system and acetylcholinesterase [47].

Later, the amyloid cascade hypothesis was proposed to explore the mechanism of AD and has been the gold-standard-beta-amyloid dogma for almost 30 years [48] and has become one of the most dominant research focuses conducted in academia and the pharmaceutical industry.

The typical hallmarks of AD-synaptic dysfunction and senile plaques—are consequences of the production, oligomerization and self-aggregation of beta-amyloid (Aβ). Amyloid precursor protein (APP) is degraded via the non-amyloidogenic (catalyzed by α-secretase and γ-secretase) and amyloidogenic pathways, where the degradation by β-secretase (BACE-1) and γ-secretase generate Aβ species. The Aβ species are composed of 37–49 amino acid residues, with the major species being Aβ40, whereas the major type from the minor species is Aβ42. Aβ40 is the more common metabolite and may actually be anti-amyloidogenic [49], whereas Aβ42 and other longer peptides are highly self-aggregating and lead to profound Aβ deposition [50,51]. Studies of AD-causing mutations in APP, presenilin 1 (PSEN1), and presenilin 2 (PSEN2) genes demonstrate that the vast majority of these mutations alter APP processing in a manner that either increases the absolute or relative levels of Aβ42 [52].

The key histopathological hallmark of AD is the senile plaque-intracellular neurofibrillary tangles in the brain. Tangles are composed of paired helical filaments and straight filaments which are mainly caused by hyperphosphorylated tau protein [53]. The tau protein hypothesis of AD is based on hyperphosphorylation of the tau protein (from the 2–3 to the 5–9 phosphate groups) by the threonine-serine kinase, GSK-3β. Such hyperphosphorylated tau protein is separated from microtubules and subsequently aggregates into insoluble intracellular neurofibrillary tangles which ultimately cause cell death [54,55]. GSK-3β is a pivotal kinase in neurodevelopment and involved in both physiological and pathological aging. In the non-amyloidogenic pathway, GSK-3β may down-regulate the activity of the α-secretase complex through inhibition of metalloproteinase (ADAM) activity [56] and regulates Aβ production by interfering with APP cleavage at the γ-secretase complex [57]. In turn, in the amyloidogenic pathway, GSK-3β inhibition reduces BACE1-mediated cleavage of APP through a nuclear factor kappa-light-chain-enhancer of an activated B cell (NF-κB) signaling-mediated mechanism. This observation thus suggests that the inhibition of GSK-3β reduces Aβ pathology [58,59,60] and GSK-3β plays a key role in choline metabolism, which involves the regulation of choline acetyltransferase (ChAT) and AChE [61,62]. Further, GSK-3β has the capacity to phosphorylate several mitogen-activated protein kinases (MAPKs), thus regulating axonal stability. Inhibitors of GSK-3β provide protection from intrinsic apoptotic signaling, but potentiate that of extrinsic apoptosis [63].

GSK3β mediates an interaction between two major forms of synaptic plasticity in the brain, *N*-methyl-d-aspartate (NMDA) receptor-dependent long-term potentiation (LTP) and NMDA receptor-dependent long-term depression (LTD). LTP and LTD of hippocampal synaptic transmission represent the principal experimental models underlying learning and memory. In mouse models of AD, early impairments in synaptic transmission were caused, among other factors, by Aβ, which leads to impairment of LTP via tau protein [64]. In the normal brain, activation of GSK3β is essential for NMDA receptor-dependent LTD, and its activity can be regulated by LTP. Following the induction of LTP, there is inhibition of GSK3β activity, whereas GSK3β inhibitors block the induction of LTD. In addition, GSK-3β has been identified as a prominent regulator of inflammation via the promotion of the production of proinflammatory cytokines (interleukin-6(IL-6), IL-1β) and tumor necrosis factor (TNF), or by decreasing the production of the anti-inflammatory cytokine, IL-10 [65,66]. To conclude, GSK-3β inhibition is a popular target for small molecule compounds, mainly based on the three therapeutic approaches, namely the inhibition of phosphorylation, prevention of the aggregation of tau protein, and stabilization of microtubules.

NMDA receptor plays a crucial role in modifying major forms of synaptic plasticity, certain types of learning and memory formation, as well as consolidation of short-term memory into long-term memory under physiological conditions [67]. The glutamatergic (NMDA) hypothesis of AD is based on the observation that inhibition of the NMDA receptor would ameliorate the overall condition of AD patients. However, NMDA cannot be fully antagonized since it exerts important bio-functions in normal synaptic transmission, whereas glutamate-related excitotoxicity and cell death could be caused when NMDA receptors are overstimulated by excess glutamate [68]. Thus, establishing an optimal balance between glutamate stimulation and glutamate-related excitotoxicity is crucial to achieve the most effective treatment of AD.

Other targets for AD are the monoamine oxidase (MAO) enzymes, a group of enzymes consisting of two distinct isoforms (MAO-A and MAO-B) which, by deamination, lead to the metabolism of amine neurotransmitters (e.g., monoamine neurotransmitters). In AD patients, the activity and gene expression of MAO-A is up-regulated in different brain areas [69,70] as well as MAO-B [71]. High levels of MAOs catalyze oxidative deamination, increasing the production of hydrogen peroxide and reactive oxygen species (ROS), which are responsible for oxidative injuries and the toxic environment characteristic of neurodegeneration [72,73] and increased MAO-B levels can enhance astrogliosis in the brain [74,75].

AD may be implicated by high levels and dysregulation of Cu^2+^, Fe^2+^, Zn^2+^, and Ca^2+^, which are important biometal ions [76,77]. Cu^2+^ and Zn^2+^ are known to induce the generation of toxic Aβ oligomers by binding to Aβ peptides and influencing the Aβ aggregation pathway [78,79], the redox-active metals, Cu(I/II) and Fe(II/III) generate cytotoxic reactive oxygen species (ROS) [80]. Thus, the use of biometal chelators that can down-regulate high levels of biometals could be a potential therapeutic strategy for the treatment of AD. The serotoninergic neurotransmitter and histaminergic systems play a critical role in the regulation of the CNS. Brain functions mediated by 5-HT_4_R and 5-HT_6_R require a synergistic effect from cholinergic neurotransmission. Activation of 5-HT_4_R can enhance the release of ACh in the hippocampus, whereas 5-HT_6_R blockade enhance cholinergic neurotransmission [81,82]. Moreover, 5-HT_4_R agonists could promote the nonamyloidogenic cleavage of APP, which releases a soluble sAPPα fragment which, in contrast to Aβ, has putative neurotrophic and neuroprotective properties [83]. The selective partial 5-HT_4_ agonist, RS-67333, which is a potent cognitive and learning function enhancer, may reduce Aβ production and has a neuroprotective activity in a cellular model of AD [84,85,86]. Activation of the histamine H_3_ receptor decreases the presynaptic release of ACh, and its blockade augments the presynaptic release of Ach, resulting in improved cholinergic neurotransmission in the cortex. However, in clinical trials, the H_3_ receptor antagonists failed to achieve cognitive improvement in AD patients [87].

Impaired signaling pathways of cyclic-3′,5′- adenosine monophosphate (cAMP) and cyclic-3′,5′-guanosine monophosphate (cGMP) may contribute to the development and progression of AD. Thus, phosphodiesterase inhibitors (PDEIs), such as rolipram and roflumilast (PDE4Is), vinpocetine (PDE1I), cilostazol and milrinone (PDE3Is), sildenafil and tadalafil (PDE5Is) were found to be involved in the phosphorylation of tau, aggregation of Aβ, neuroinflammation as well as regulation of cognition, mood, and emotion processing. Despite rational arguments, the clinical data do not demonstrate efficacy of selective PDEIs in improving cognition in patients with prodromal and mild AD, and a number of these have been discontinued due to failure to meet efficacy endpoints in a phase I/II clinical trial [88,89,90]. However, the combination therapy of donepezil with cilostazol showed positive effects on patients with mild or moderate-to-severe Alzheimer’s patients [91,92]. To conclude, there are many biological targets and signalling pathways involved in AD pathology [93,94]. However, the complex interactions between them is unclear.

## 3. Multi-Target Strategy for AD

**Figure 3:**
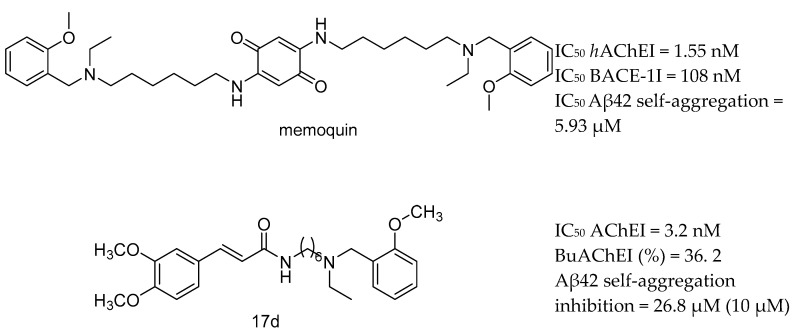
Memoquin and 17d, the most active ferulic acid-memoquin hybrid, from [97].

The complex nature of AD has led to the development of a multitarget approach in the design and development of new potential anti-AD drugs. At present, the MTDLs strategy can be divided into two main categories based on the biological targets, namely, involving AChE or distinct to AchE. The first category is mainly based on inhibitors of AChE/BuChE and β-amyloid aggregation. The success of memoquin and ferulic acid-memoquin hybrids (Figure 3), which can inhibit both AChE- and self-induced Aβ aggregation, open exciting new opportunities for MTDLs drug discovery [95,96,97].

The MTDLs involving AChE consist mainly of tacrine- or donepezil-related compounds. Tacrine and donepezil are non-competitive and reversible ChE inhibitors and alleviate neuronal degeneration caused by damage of cholinergic transmission. On the other hand, AChE has become to be regarded as an inducer to trigger the aggregation of Aβ. Interaction of a peripheral anionic site of AChE with Aβ could facilitate fibril formation. Blocking the peripheral anionic site of AChE has been identified as an effective method for inhibition of Aβ aggregation [98]. The simultaneous binding to a catalytic active and a peripheral anionic side of AChE (dual-site inhibitors) is an example of successful adaptation of an MTDLs design strategy applied in the most prevalent and dominant class of tacrine hybrids. However, various donepezil-based cholinesterase inhibitors could also show additional inhibition of Aβ aggregation, oxidative stress, and MAOs. MTDLs based on tacrine and donepezil scaffolds are described in Table 2.

### 3.1. AChE and BACE-1 Inhibitors

The analysis of the SciFinder database showed that there were almost 567 articles (original and review) related to combining the functionality of AChE drugs and BACE-1 inhibitor pharmacophores over the last decade. MTDLs which contain a pharmacophoric moiety derived from tacrine are potent BACE-1 inhibitors, inhibitors of tau-protein aggregation and possess additional properties (antioxidative, neuroprotective or metal chelating ability) [122,123,124,125,126,127] as well as another major chemical group consisting of donepezil-related compounds [121,128,129,130,131,132,133,134,135,136]. Moreover, miscellaneous compounds based on the cyanopyridine, quinazoline, quinoline, benzo-chromene, pyrimidinimine, and thiazole cores are inhibitors of both AChE and β-amyloid aggregation and some of them possess antioxidative properties and inhibitory potency against BACE-1 [137].

**Figure 4:**
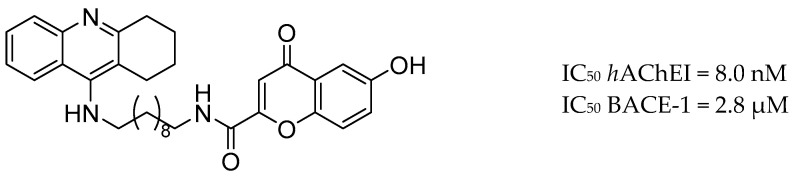
The most active tacrine and 4-oxo-4*H*-chromene hybrid from [138].

A family of tacrine and 4-oxo-4*H*-chromene hybrids was reported by Fernandez-Bachiller and co-workers. The most promising compound (Figure 4) exhibited potent inhibition activity against human AChE and BACE-1, antioxidant activity (1.3-fold more potent than a vitamin E analogue–trolox) and good CNS permeability [138].

**Figure 5:**
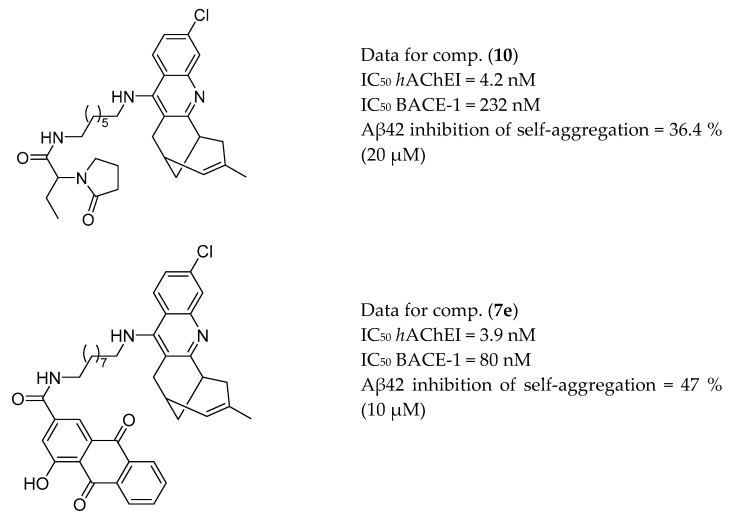
The most active levetiracetam-huprine and rhein-huprine hybrids from [125,140].

Muñoz-Torrero et al. synthesized a series of heptamethylene-linked levetiracetam-huprine and levetiracetam-6-chloro-tacrine hybrids with potent inhibitory activities against human AChE and BACE-1 and Aβ42 anti-aggregating activity. The most interesting compound, (**10,** Figure 5), reduced the frequency of spontaneous convulsions, prevented memory impairment and reduced the Aβ burden in the cortex of APP/PS1 mice [125]. The presented hybrid protects transgenic mice from cognitive deficits and, thereby, can be considered a multifunctional disease-modifying anti-Alzheimer agent. Additionally, the group has reported on second-generation anti-AD rhein-huprine hybrids with potent inhibitory activities against human AChE and BACE-1 and Aβ42 anti-aggregating activity [139]. The most potent compound (**7e**, Figure 5), after intraperitoneal administration in APP-PS1 transgenic mice, showed a central effect in lowering soluble Aβ [140].

**Figure 6:**
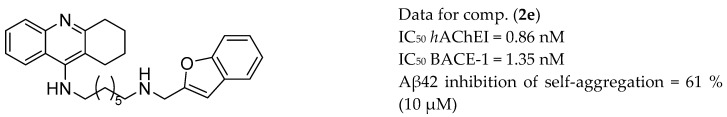
The most active tacrine–benzofuran hybrid from [126].

Zha et al. designed and synthesized novel tacrine–benzofuran derivatives containing amino or amido linkages. Most hybrids exhibited good inhibitory activities on AChEs and β-amyloid self-aggregation. The most promising compound (**2e**, Figure 6) showed an interesting profile as a subnanomolar selective inhibitor of *h*AChE and a good inhibitor of both β-amyloid aggregation (hAChE- and self-induced, 61.3% and 58.4%, respectively) and *h*BACE-1 activity. This hybrid also exhibited lower hepatotoxicity than tacrine and in vivo studies confirmed its ability to penetrate the BBB. Next, in scopolamine-induced memory impairment studies in knock-out mice, the hybrid significantly ameliorated memory performance of mice in a Morris water maze test [126].

### 3.2. AChE and GSK-3β Inhibitors

**Figure 7:**
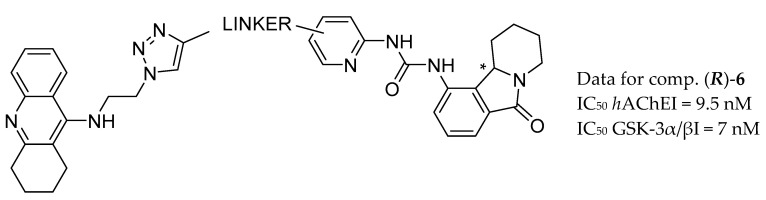
Tacrine and valmerin hybrids from [100].

Two important AD-related targets influencing both ACh concentration modulation and tau protein phosphorylation, are AChE and GSK-3β. MTDLs possessing inhibitory potency for both enzymes have been rarely reported so far. Potent AChE and GSK-3β dual-target inhibitors were obtained by hybridizing the pharmacophores of GSK-3βI with AChEI. Thus, tacrine was incorporated at the thiazolyl ring of potent GSK-3βI with an appropriate linker. The most promising hybrid showed the most interesting profile as a nanomolar dual enzyme inhibitor, good inhibitory effect on Aβ self-aggregation, inhibition of tau protein hyperphosphorylation, and significant in vivo cognitive improvement in mice tests [141]. Hui et al. linked tacrine (AChEI) with phenothiazine via an alkylenediamine-type spacer. Phenothiazine is a core of methylthioninium chloride, also known as methylene blue or Rember^®^, which has been shown to reduce tau levels in vitro and in vivo in several different mechanisms of action [142,143]. In addition, an appropriate linker containing a 1,2,3-triazole moiety for two scaffolds, tacrine and valmerin (GSK-3α/β), was designed based on molecular docking and crystallography [100] (Figure 7).

### 3.3. AChE and MAO Inhibitors

**Figure 8:**
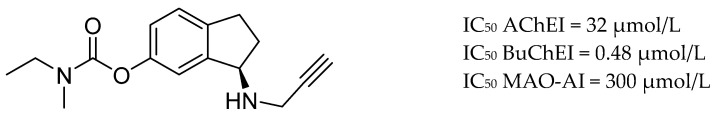
Ladostigil.

MTDLs strategies involving the AChE and MAOs targets in the SciFinder data base showed over 400 articles (original and review) related to combining the functionality of AChE drugs and MAOIs [144,145,146,147,148,149,150,151]. Ladostigil (Figure 8) was designed on the basis of the structures of rivastigmine and rasagiline. However, clinical trials revealed that the drug failed in its primary endpoint of curbing progression from mild cognitive impairment to AD [152,153]. Homo isoflavonoid Mannich-based derivatives, the selective dual inhibitors of AChE and MAO-B, exhibited Aβ(1–42) aggregation inhibitory efficacy with antioxidant activity and biometal chelating ability [154].

### 3.4. AChE Inhibitors and NMDA Antagonists

**Figure 9:**
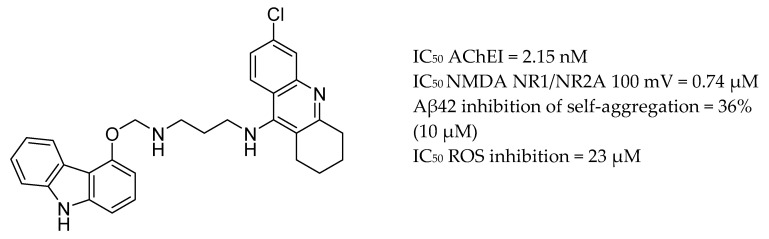
Carbacrine.

The degenerative process of cholinergic neurons in AD are implicated by excessive activation of the NMDA receptor. Thus, NMDA receptor antagonists can confront neurodegeneration and AChEIs can recover memory and cognition. Namzaric was approved in 2015 for the treatment of AD, using a once-daily fixed-dose drug combination comprised of memantine hydrochloride and donepezil hydrochloride. The first rationally-designed multifunctional ligand, carbacrine, was obtained by integrating synergistic fragments of the chloro-substituted tetrahydroacridine moiety of 6-chlorotacrine and carbachol as the starting lead components [155]. Carbacrine showed dual inhibitory activity against AChE and the NMDA receptor, blocking in vitro Aβ self-aggregation and aggregation mediated by AChE, antagonizes NMDA receptors, and reduces oxidative stress [156] (Figure 9).

**Figure 10:**
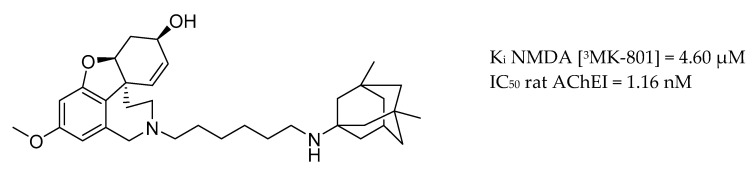
Memagal.

More recently, a new class of multi-target compounds that combine the structures of galantamine and memantine have been designed. The most potent drug candidate, memagal (Figure 10), showed a remarkable inhibitory potency against AChE and NMDA receptor inhibition which was tested by a [^3^H] MK-801 binding assay.

Optimization of the dimebion structure (a multi-target antihistamine drug) led to compounds with a multi-target profile against both the AChE and NMDA receptors and self-induced Aβ aggregation inhibitory potency [157]. Makhaeva and co-workers fused dimebon with phenothiazine, and obtained compounds with dual BChE and NMDA receptor-inhibition potency [158].

### 3.5. AChE Inhibitors and 5-HTR

**Figure 11:**
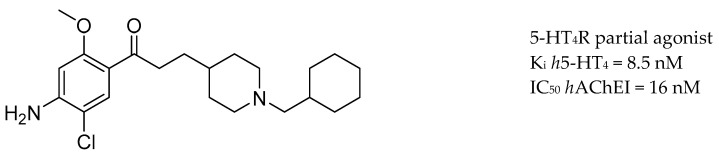
Donecopride.

Modulating neurotransmission of ACh and 5-HT could be achieved through 5-HT_4_ and 5-HT_6_ receptors (5-HTR). Activation of 5-HT_4_R can enhance the release of ACh in the hippocampus, 5-HT_4_R agonists can promote the nonamyloidogenic cleavage of APP, forming neurotrophic human soluble amyloid precursor protein α (sAPP-α) fragments and decrease Aβ secretion in primary neurons [81]. Moreover, 5-HT_6_R antagonists are thought to have the ability to enhance cholinergic neurotransmission [82,159,160]. RS67333, a partial 5-HT_4_R agonist well-known for its precognitive effect, showed a synergistic effect with donepezil and lowered Aβ levels by directly inhibiting the activity of AChE [161]. In addition, the key pharmacophores of RS67333 and donepezil were merged into a single novel chemical entity, leading to the discovery of donecopride [86], a dual-action AChEI and 5-HT_4_ receptor agonist. Donecopride (Figure 11) stimulates the nonamyloidogenic 5-HT_4_ receptor-mediated cleavage of APP and has a greater potency in promoting neurotrophic sAPP-a release compared with RS67333.

### 3.6. AChE Inhibitors and H_3_R

**Figure 12:**
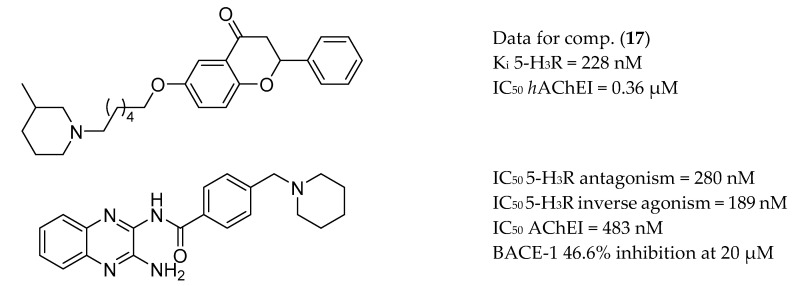
Hybrid involving AChE and H_3_R from [165,166].

The H_3_ histamine receptors (H_3_R) regulate the release of histamine, acetylcholine and other monoamines. Activation of H_3_R decreases the presynaptic release of ACh, and its blockade augments the presynaptic release of ACh and improves cholinergic neurotransmission in the cortex. Evaluation of H_3_R antagonists or inverse agonists revealed their promnesic properties and ability to modulate learning by improving memory consolidation [162]. One of these antagonists or inverse agonists of H_3_R, pitolisant, has been approved for clinical use in patients with narcolepsy [163,164]. Thus, synergistic effects on up-regulating synaptic levels of ACh could be achieved by involving AChE and H_3_R. Bajda et al. applied docking-based virtual screening for novel multifunctional compounds in a non-imidazole histamine H_3_R ligand library. The most promising derivatives combined the flavone moiety via a six-carbon atom linker with a heterocyclic moiety, such as azepane, piperidine or 3-methylpiperidine (Figure 12). Compound **17** showed the highest inhibitory activities toward cholinesterases as well as well-balanced potencies against H_3_R and both ChE enzymes [165]. Huang and co-workers reported a series of compounds with a quinoxaline scaffold that showed related inhibitory activities against AChE, H_3_R, and BACE-1 targets [166] (Figure 12).

### 3.7. AChE Inhibitors and Biometal Chelators

**Figure 13:**
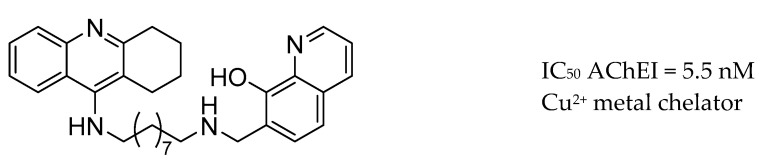
Hybrid of tacrine-clioquinol.

Starting with the hypothesis that biometal chelators that can down-regulate high levels of biometals could form a potential therapeutic strategy for the treatment of AD, Fernandez-Bachiller and co-workers reported a hybrid fused with tacrine and the metal chelator drug, clioquinol (Figure 13) [167].

**Figure 14:**
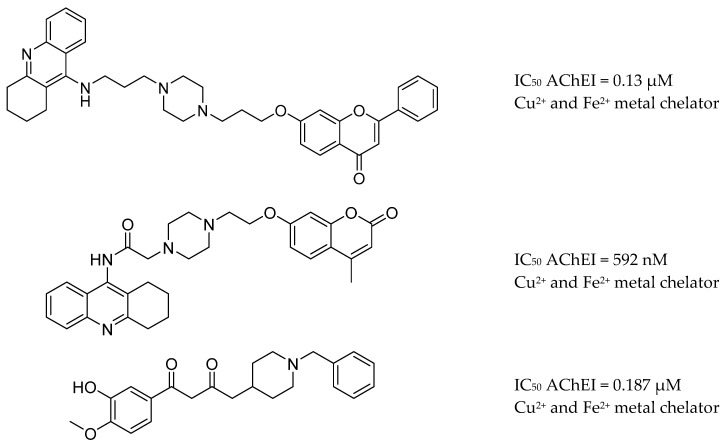
Hybrids of tacrine-flavone and donepezil-curcumin from [168,169,170].

In turn, hybrids of tacrine and a flavone, namely coumarin, chromone or donepezil, and curcumin, exhibited a significant ability to inhibit AChE. Additionally, hybrids of tacrine and a flavone, such as coumarin, chromone or donepezil and curcumin, exhibited significant β-amyloid-reducing and metal (Cu^2+^ and Fe^2+^) chelating properties [168,169,170] (Figure 14).

**Figure 15:**
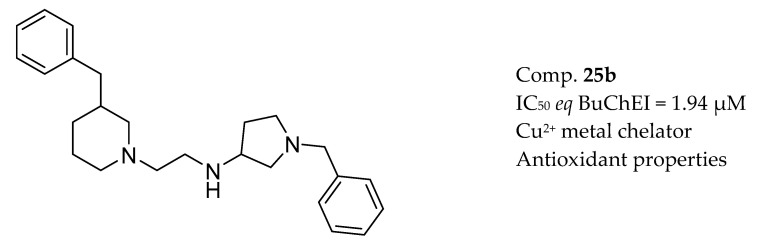
Inhibitor of BuChE with antioxidant and metal-chelating properties from [172].

Wichur et al. designed and synthesized novel 1-benzylpyrrolidine-3-amine-based BuChE and BACE-1 inhibitors with activities towards Aβ and tau protein aggregation, as well as concomitant antioxidant and metal-chelating properties [171] (Figure 15).

### 3.8. BACE-1 and GSK-3β Inhibitors

**Figure 16:**
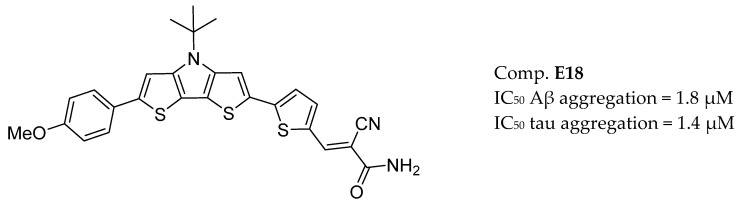
Oligo heteroaromatic hybrid with BACE-1 and GSK-3β inhibitory activity from [172].

Even though the importance of AChE in the AD signalling network is prominent, MTDLs strategies are also under consideration in the treatment of specific and multiple protein pockets of AD-associated targets. Anti-amyloid based strategies not only focus on Aβ alone, but also on effective modulation of the robust network of Aβ-mediated events. Thus, dual inhibitors of BACE-1 and GSK-3β show promise as drug candidates based on the inhibition of Aβ and tau protein aggregation [172] (Figure 16).

**Figure 17:**
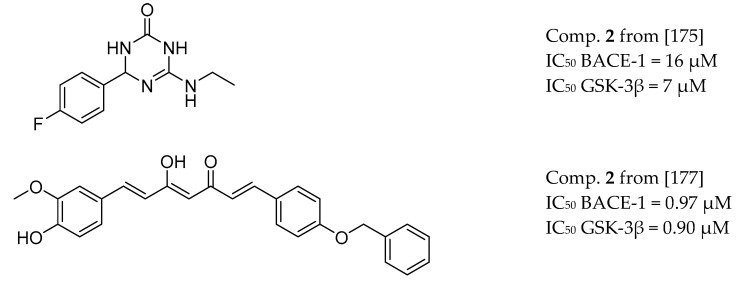
BACE-1 and GSK-3β inhibitors.

Table 1. GSK-3β dual inhibitors was obtained based on a 3,4-dihydro-1,3,5-triazin-2(1*H*)-one skeleton. Subsequently, triazinones (6-amino-4-phenyl-3,4-dihydro-1 and 3,5-triazin-2(1*H*)-ones), with a critical pharmacophore comprising the cyclic amide group and guanidino motif, have been shown to constitute a promising class of multifunctional fragments able to modulate tau and amyloid cascades simultaneously (Figure 17) [173]. Bottegoni et al. reported an optimized virtual screening method to identify fragments with multifunctional activities against BACE-1 and GSK-3β [174]. Another approach described a series of dual inhibitors targeting BACE-1 and GSK-3β, based on curcumin derivatives [175] (Figure 17).

### 3.9. Other Targets

The approved antifungal drug, clioquinol, with an 8-hydroxyquinoline scaffold, was shown to extract metal ions from extracellular Aβ aggregates. Thus, a series of novel hybrids fused with the main pharmacophores of selegiline and clioquinol, showed MAO-B inhibitory potency, antioxidant activity, biometal chelating ability, and effective inhibition against Cu(II)-induced Aβ aggregation [176].Clioquinol was then fused with the PDE9A inhibitor, PF-04447943, and the resulting hybrid showed inhibitory potency against PDE9 with high selectivity over other PDEs, notable Cu^2+^-induced Aβ aggregation inhibition, and favorable blood–brain barrier (BBB) permeability [177]. New perspectives were presented by novel hybrids endowed with anti-inflammatory and anticholinesterase activity via triple-targeting properties. Hybrids obtained through the merger of triazoles and thiosemicarbazides simultaneously impact on cholinesterases, cyclooxygenase-2 (COX-2) and 15-lipoxygenase (15-LOX), and have emerged as promising new hits [178].

Epigenetics involves the study of heritable and reversible changes in gene expression that cannot be explained by changes in the sequence of bases in genomic DNA but through covalent modifications to the cytosine residues of DNA, covalent chemical modifications in histones and chromatin remodeling, and noncoding RNAs. Such transcriptional dysregulation plays an important role in the progression and development of AD mainly through transcription of immediate early genes (IEGs), important for neuronal plasticity, memory and behavior [179,180]. Thus, there are interesting targets concerning epigenetics, especially lysine (K)-specific demethylase 1A (KDM1A or LSD1) and histone deacetylase (HDAC). KDM1A is a flavin adenine dinucleotide (FAD) dependent amine oxidase that acts primarily as a histone demethylase and has been implicated in the control of IEG transcription. HDAC is involved in the deacetylation of histone proteins which promote heterochromatin, leading to silenced gene transcription and expression. KDM1A and HDAC inhibitors have a promising therapeutic potential in recovering memory defects and cognitive disabilities through eliminating these diverse molecular and cellular functions of HDAC enzymes in the brain.

**Figure 18:**
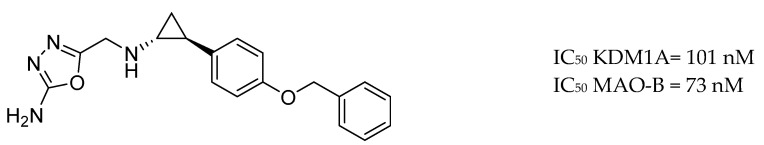
Vafidemstat (ORY-2001).

Vafidemstat (ORY-2001) (Figure 18), an oral, brain penetrating, dual KDM1A/MAO-B inhibitor active at doses suitable for long-term treatment, corrects memory deficit in the Senescence Accelerated Mouse Prone 8 (SAMP8) model for accelerated aging and Alzheimer′s disease. Moreover, multiple genes modulated by ORY-2001 are differentially expressed in late onset Alzheimer’s disease. Vafidemstat is currently in multiple Phase IIa studies [181].

**Figure 19:**
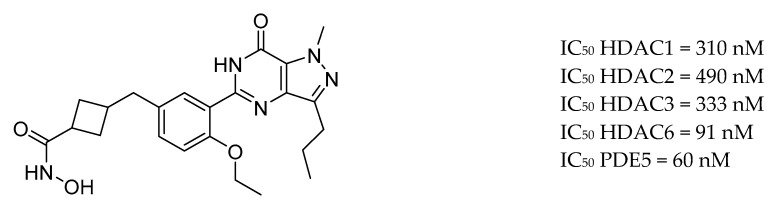
CM-414—first-in class dual inhibitor of HDAC and PDE5.

Cuadrado-Tejedor et al. reported CM-414, a first-in class small-molecule that acts as a dual inhibitor of HDAC and PDE5 (Figure 19). Inhibition of PDE5 induces an increase in the activation of cAMP/cGMP- responsive element-binding protein (CREB) which combined with moderate HDAC class I inhibition, leads to efficient histone acetylation. CM-414 rescued the impaired long-term potentiation evident in hippocampal slices from APP/PS1 mice, diminished brain Aβ and tau phosphorylation (pTau) levels in chronic treatment of Tg2576 mice [182].

**Figure 20:**
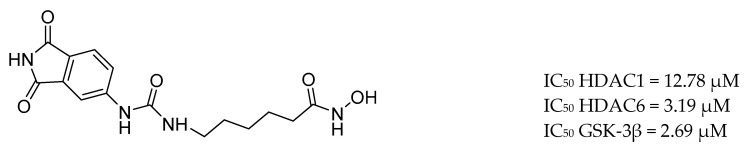
*GSK-3β/HDAC dual inhibitor* [184].

The combined treatment of GSK-3β and HDAC inhibition induces synergistic neuroprotective effects in various in vitro and in vivo models of neurological diseases. De Simone et al. discovered the first-in-class GSK-3β/HDAC dual inhibitor as disease-modifying agent for AD (Figure 20). Compound **11** induces an increase in histone acetylation and a reduction of tau phosphorylation. Moreover, it promotes neurogenesis and displays immunomodulatory effects. It is nontoxic and protective against H_2_O_2_ and 6-OHDA stimuli in SH-SY5Y and in CGN cell lines, respectively [183].

**Figure 21:**
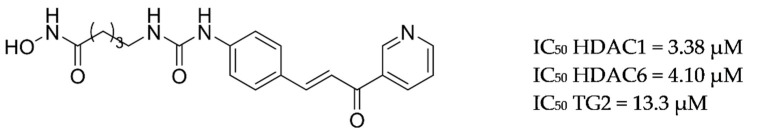
Dual TG2 and HDAC inhibitor from [185].

Transglutaminase 2 (TG2) is the most studied and expressed isoform of the TG family which is important in cross-linking proteins in Alzheimer’s disease. Next, the genetic elimination of TG2 in Huntington’s disease (HD) mice models ameliorated the symptoms and restored the message and protein levels of brain-derived neurotrophic factor (BDNF). Moreover, the inhibition or genetic suppression of TG2 halts oxidative stress-induced cell loss in cortical neurons [184,185]. Thus, neuroprotective effects can be achieved by the TG2 and HDAC inhibition, which synergistically protects against toxic stimuli mediated by glutamate. The most active compound (**3**) [(*E*)-*N*-hydroxy-5-(3-(4-(3-oxo-3-(pyridin-3-yl)prop-1-en-1-yl)phenyl)thioureido)pentanamide] inhibit TG2 and HDACs both in vitro and in cell-based assays (Figure 21). Furthermore, this nontoxic compound protects cortical neurons against toxic insults induced by glutamate [185].

In summary, a multitarget strategy for AD is emerging, involving a broad spectrum of potential therapeutic strategies from modulating neurotransmission, tau-based therapies, amyloid based therapies, modulating intracellular signalling cascades, oxidative stress reduction, modulation of cellular calcium homeostasis, to anti-inflammatory therapy. Research in the field of application of MTDLs strategy in the field of AD has been extensively reviewed elsewhere [118,186,187,188].

## 4. Physicochemical Properties of MTDLs for AD

MTDLs are designed by combining two (or more) pharmacophoric structural scaffolds, thus physicochemical properties such as molecular weight, solubility, permeability and the ability of MTDLs to permeate cell membranes have to optimized for absorption and distribution within the body [189]. The ‘Lipinski rule of five’ (RO5) [190] defined the optimal physicochemical properties for perorally administered drugs, which are preferred for perspective long-term treatment of AD patients. RO5 postulated desirable physicochemical property space by setting up limits for molecular weight < 500Da, logarithm of partition coefficient (log*P*; a value classifying the lipophilicity of a compound) < 5, number of hydrogen bond donors (HBDs)< 5, and number of hydrogen bond acceptors (HBAs)< 10. Although RO5 indicates possibility of absorption of drug after oral administration, the rule is not specific for CNS drugs. It turns out that AD drugs need to fulfil strict physicochemical criteria defined for blood–brain barrier (BBB) penetration by transcellular passive diffusion, which is typical for most CNS drugs. Blood-to-brain influx is mediated by the receptor for advanced glycation end products (RAGE). The BBB efflux pumps are transmembrane P-glycoprotein (P-gp), which mediate brain-to-blood transport. Cordon-Cardo et al. first suggested that P-gp might play a role in vivo in limiting brain penetration of xenobiotics [191]. The high expression of P-gp is one of the main reasons that a lot of lipophilic drugs could not penetrate CNS for brain disease therapy.

Extensive studies on physicochemical properties for CNS drugs laid out that molecular weight should not exceed 430–450 Da, log*P* values should be < 4 and the number of HBD and HBA must be < 2 and < 7, respectively [192]. Next, characterisation has been completed with topological polar surface area (TPSA), Log D and acid dissociation constant (p*K*_a_). TPSA counts the surface sum over all polar atoms in molecule, and for most CNS drugs to be < 70 Å^2^. LogD is a pH dependent lipophilicity indicator displaying the distribution of a chemical compound between the lipid and aqueous phase. For CNS drugs logD is expected to be <3. The strength of acidic functional groups in the molecule expressed by pKa values for CNS drugs should be 7–9. For streamlining the design of proper physicochemical properties of MTDLs for AD, several powerful predicting techniques, such as BBB-score and the CNS multiparameter optimization approach, have been developed. However, the most of MTDLs have been tested in a parallel artificial membrane permeation assay (PAMPA), which experimentally determine permeability values which would enable them to cross the BBB by passive diffusion [125,127,132,193,194]. The current chemical methods for crossing the BBB could be divided into chemical modification of the drug to form a prodrug, coupling the drugs with mannitol or aromatic substances or using appropriate chemical drug delivery system or drug carrier with the ability to cross BBB [195].

## 5. Perspectives for MTDLs in the Treatment of AD

At present, only symptomatic treatments exist for AD, namely 3 cholinesterase inhibitors and memantine, which mainly block the progression of the disease and are supposed to interfere with the pathogenic steps responsible for the clinical symptoms. AD is a complex disease, thus the rational way for searching for pathway-target-drug-disease relationships seemed to be a genome-based analysis [196,197]. Kwok et al. [198] used a gene-based test for the genetic validation of potential AD drugs, to provide an initial screening tool for the identification of drugs that are unlikely to be successful. This study, however, provides no evidence that any approved or investigational AD drugs target products of genes strongly associated with late-onset AD, which might explain the lack of efficacy to date.

MTDLs toward AD have to confirm their simultaneous multitarget engagement in both in vitro and in vivo assay systems. Thus, the use of cellular models (cell-screening systems) of AD, based on human induced pluripotent stem cell (hiPSCs) technologies could change preclinical research [199]. In addition, the animal models currently available cannot fully reflect the multifactorial nature of human AD for multiple ligand testing. Age-dependent neurodegeneration mouse models that mimic AD rely mostly on the neuronal overexpression of human proteins carrying a familial AD-causing mutation in presenilin, which increases Aβ42 production and oligomerization [200,201]. In fact, with respect to neurodegenerative diseases generally, mouse mutations corresponding to human disease-linked mutations rarely result in neurodegenerative phenotypes, mostly because age is the single greatest risk factor for neurodegeneration, and mice have much shorter lifespans than humans.

Up to 2019, over 2000 clinical trials in AD drug development had been reported in which various hypotheses for AD were tested. Liu et al. [25] reported that the amyloid hypothesis was the most heavily tested (22.3% of trials), with the neurotransmitter hypothesis being the second most tested (19.0% of trials). In turn, a systematic review of the AD drug development pipeline in 2019, showed that there were 132 agents in clinical trials and 90 agents in trials targeting cognitive enhancement, and a further 14 trials intended to treat neuropsychiatric and behavioural aspects of AD [202]. Results suggested that there is a conceptual supremacy for disease-modifying therapies, as opposed to symptomatic-disease approaches [48]. From 96 agents reported in disease modification trials, 40% have amyloid as the primary target, or as one of several effects, whereas seven small molecules and 10 biologics have tau as a primary or combination, target (18%). Until recently, AD drug development has been largely focused on beta amyloid plaques and tau tangles in the brain. These attempts have yielded less successful results than had been anticipated. Currently, some of the most novel non-amyloid approaches were put to the discovery and development of drugs for Alzheimer’s and related dementias. Firstly, repurposing an existing drug accelerates the drug development timeline. An example of this is the repurposing of a Parkinson’s drug, rasagiline, that holds promise for slowing the progression of Alzheimer’s disease in patients with mild cognitive impairment [203]. In addition, new drug candidates could restore lost cognitive function and lead to neuroprotective therapies for AD and other forms of dementia [204].

The diversification of targets and the entry of combination therapies into the potential therapeutic pipeline is particularly noticeable between phase 3 and 2 of clinical trials. The using of new biomarkers allow early assessments of the impact of candidate interventions on disease biology [202], however there is lack of accurate biomarkers to identify and track the progression of AD, which has slowed therapeutic development in tauopathies.

So far, a wide range of new biological targets are under consideration as microglial targets (Table 3). Microglia are a class of innate immune cells (macrophages) within the CNS. Microglia fulfil a number of varied roles within the CNS including the immune response, maintenance of homeostasis, extracellular signalling, phagocytosis, antigen presentation and synaptic pruning [205]. In AD, microglia reaction was initially thought to be incidental and triggered by Aβ deposits and dystrophic neurites. Moreover, recent genome-wide association studies have established that the majority of AD risk loci are found in or near genes that are highly and sometimes uniquely expressed in microglia [206]. On the other hand, a multitude of independent sources have suggested that neuroinflammation contributes to the pathogenesis of AD. Therefore, a hypothesis has been proposed that microglia could be critically involved in the early steps of AD and microglial targets are now being considered as the next important potential therapeutic targets.

It is also worth mentioning the innovative nanotechnology-based approaches in the treatment of AD, an approach that is seen as a great hope for developing new treatment strategies. The achievements of nanotechnology are particularly encouraging because they provide many benefits while overcoming the limitations of conventional formulations.

Recently, Chen et al. [210] reported the preparation of a methylene blue loaded multifunctional nanocomposite. The constructed nanocomposite has ultra-small ceria nanocrystals and iron oxide nanocrystals assembled onto the surface of mesoporous silica nanoparticles, methylene blue loaded into its pores and, additionally, a tau tracer, Amino-T807, grafted onto the surface. The adopted strategy allowed for the achievement of a promising synergistic effect as a result of ROS scavenging ability of ultra-small ceria nanocrystals, ameliorating mitochondrial oxidative stress-induced damage and use of a tau aggregation inhibitor, methylene blue, which could be released in neurons to prevent hyperphosphorylated tau aggregation. On the other hand, Guo et al. [211] designed and optimized a novel fusion peptide (TPL) comprising a BBB-penetrating peptide, TGN, and a neuron binding peptide, Tet1, through a four-glycine linker. Interestingly, TPL-modified nanoparticles led to an increase in BBB-penetration and neuron targeting efficacy, in comparison to the nanoparticles co-decorated with the two mono-ligands. Moreover, after the encapsulation of a neuroprotective peptide, NAP, the TPL nanoparticles reduced the intracellular ROS, showed protection of microtubules against Aβ25-35-induced toxicity, and rescued the OA-induced tau aggregation and neuronal apoptosis.

Another interesting study concerns the development of a polymeric micelle drug delivery system consisting of the RAGE targeting peptide (Ab) derived from Aβ protein, an amphiphilic polymer with ROS responsiveness and scavenging ability, and curcumin, a natural compound which has been described to target to Aβ aggregation [212]. Such a type of nanosystem has the ability to accumulate in the AD brain and initiate subsequent microglia-based microenvironment modulation. More importantly, it is possible that multiple targets within the AD microenvironment could be controlled to reeducate the hyperactive microglia and protect damaged neurons. The examples listed above confirm the need for further intensive studies to obtain better clinical translation of multifunctional nanosized drug delivery systems.

## 6. Conclusions

The increasing number of potential biological targets for AD raises the question of how to find meaningful combinations of targets for an MTDLs approach. A pioneering study of a human pharmacology interaction network revealed relationships between proteins in chemical space and assessed the polypharmacology profile of ligands [213]. Next, a computational model to map the polypharmacology network was assembled by connecting targets according to the structural similarity of their binders (similarity ensemble approach, SEA) [214]. Thus far, the computationally driven multitarget hit discovery has been successfully applied to the discovery of MTDLs combining galantamine and memantine [215] and triazinones as BACE-1 and GSK-3β inhibitors [216]. Besnard et al. outlined a strategy for the automated design of MTDLs for the optimization process of multitarget hits [217].

Modern drug discovery has the tools to screen millions of compounds or fragments to determine potential association with AD. Oset-Gasque and Marco-Contelles have proposed that there is a need to find universal and polyvalent pharmacophoric groups able to modulate diverse receptors or enzymatic systems [218]. For novel MTDL hits against AD, two applicable strategies have been used, namely, the framework combination approach or fragment-based drug discovery (FBDD) [219,220,221]. Meanwhile, Prati et al. proposed the adoption of both strategies, in parallel [222]. The identification of a high-quality starting hit is crucial for next steps towards lead optimization. A high-quality starting hit is characterized by a balanced modulation of several targets, but it is still a challenge to choose the right combination of targets. The main goal of MTDL design is to choose such combinations of targets that can provide a superior therapeutic effect and side-effect profile, compared to the action of a selective ligand on its own. It requires a good understanding of target-disease associations, pathway-target-drug-disease relationships and adverse events profiling. However, the complex interactions among potential AD targets is complicated and the precise mechanisms are unclear. The application of a big-data research approach should be able to identify the biological pathways and key molecules involved in the aetiology of AD through the generation and analysis of large-scale transcriptome, epigenetic, proteome, and clinical data, thereby providing a rational and validated basis for extending the MTDL approach in AD [223].

## Figures and Tables

**Table 1:** Drugs approved for AD therapy.

Drug Structure	Targets	Therapeutic Effects
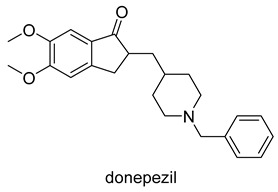	-AChE inhibitor -5-HT_2A_ inducer -ChE inducer -NOS inhibitor/inducer -TNF inhibitor -IL-1β inhibitor/inducer -NMDAR downregulator	-selectively and reversibly inhibits AChE -improves the cognitive and behavioral signs and symptoms of AD -neuroprotective
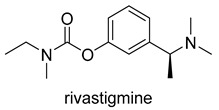	-AChE inhibitor -ChE inhibitor	-parasympathomimetic and a reversible cholinesterase inhibitor -inhibits both BuChE and AChE -enhances cholinergic function
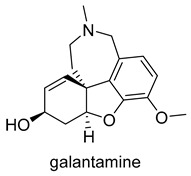	-AChE competitive and reversible inhibitor -AChR subunit alpha-7 allosteric modulator -N AChR allosteric modulator -ChE inhibitor	-enhances cholinergic function -improve cognitive performance in AD -not considered as a disease-modifying drug
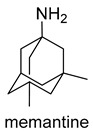	-NMDAR uncompetitive (open-channel) antagonist -Alpha-7 nicotinic cholinergic receptor subunit antagonist	-inhibits calcium influx into cells that is normally caused by chronic NMDAR activation by glutamate -enhances neuronal synaptic plasticity

**Table 2:** Examples of MTDLs based on tacrine and donepezil scaffolds.

Scaffold	Lead Fragment	Targets	Ref.
Tacrine	xanomeline, iperoxo-fragment	AChE Is and MRs	[99]
	valmerin	AChE Is and GSK-3α/βIs	[100]
	deferasirox	AChE Is and metal chelators	[101]
	indole-3acetic acid	AChE/BuChE Is	[102]
	pulmonarin B	AChE/BuChE Is	[103]
	d-xylose, d-ribose, d-galactose	AChE/BuChE Is	[104]
	acridine	AChE/BuChE Is	[105]
	1,2,3-thiadiazole	AChE/BuChE Is	[106]
	ferulic acid	AChE/BuChE Is	[107]
	flavonoid quercetin	AChE/BuChE Is and metal chelators	[108]
	quinolone carboxylic acids	AChE/BuChE Is, M_1_R Is	[109]
Donepezil	hydroxyphenyl-benzimidazole	AChE Is and metal chelators	[110]
	hydroxybenzimidazole	AChE Is and metal chelators	[111]
	butylated hydroxytoluene hybrids	AChE Is, MAO-B Is, antioxidants	[112]
	trolox	AChE Is, MAO-B Is, metal chelators	[113]
	chromone	AChE Is, MAO-B Is	[114,115]
	chalcones	AChE/BuChE Is	[116]
	2-acetylphenol	AChE/BuChE Is, MAO-A/B Is, metal chelators	[117]
	deoxyvasicinone	AChE Is and BACE-1 Is	[118]
	introduction of amide bonds	AChE Is and BACE-1 Is	[119]
	conjugation of benzylpiperidine moiety benzimidazole or benzofuran	AChE Is, antioxidant, metal chelators	[120]
	melatonin	AChE/BuChE Is, antioxydants, metal chelators	[121]

**Table 3:** Emerging microglial targets in AD based on [205,206,207,208].

Pathway	Targets	Evidence
Purinergic signalling	-ionotropic P2XRs -metabotropic P2Y -adenosine P1Rs -P2YR-dependent calcium signaling	-P2 × 7R is up-regulated in AD -P2Y2R protective role -P2Y6R—small molecule from GliaCure claims to promote microglial phagocytosis through binding of the microglial purinergic P2Y6 receptor [209] -P2Y12 plays an important role in homeostatic microglia
NOD-like receptor family pyrin domain containing 3 (NLRP3)	-triggers TREM2 receptor expressed in myeloid cells 2	-NLRP3 inflammasome activation is a pathophysiological pathway in AD
Toll-like receptors (TLRs)	-TLR2 -TLR4	-triggers the neuroinflammatory response -downstream TLR signalling through NFκB, activator protein 1 and IFN regulatory factor (IRF) pathways lead to proinflammatory gene transcription
microglial fractalkine receptor	-CX3CL1/CX3CR1	-CX3CL1 exerts an inhibitory signal, maintaining microglia in a resting state
Receptor- interacting serine/threonine-protein kinase 1 (RIPK1)	-RIPK1 inhibitor	-enzyme downstream of TNFα signalling, has been shown to mediate microglial responses in AD
